# A worldwide systematic review and meta-analysis of bacteria related to antibiotic-associated diarrhea in hospitalized patients

**DOI:** 10.1371/journal.pone.0260667

**Published:** 2021-12-08

**Authors:** Hamid Motamedi, Matin Fathollahi, Ramin Abiri, Sepide Kadivarian, Mosayeb Rostamian, Amirhooshang Alvandi

**Affiliations:** 1 Department of Microbiology, School of Medicine, Kermanshah University of Medical Sciences, Kermanshah, Iran; 2 Student Research Committee, School of Medicine, Kermanshah University of Medical Sciences, Kermanshah, Iran; 3 Fertility and Infertility Research Center, Health Technology Institute, Kermanshah University of Medical Sciences, Kermanshah, Iran; 4 Infectious Diseases Research Center, Health Institute, Kermanshah University of Medical Sciences, Kermanshah, Iran; 5 Medical Technology Research Center, Health Technology Institute, Kermanshah University of Medical Sciences, Kermanshah, Iran; Rabin Medical Center, Beilinson Hospital, ISRAEL

## Abstract

**Introduction:**

Antibiotic-associated diarrhea (AAD) is a major hospital problem and a common adverse effect of antibiotic treatment. The aim of this study was to investigate the prevalence of the most important bacteria that cause AAD in hospitalized patients.

**Materials and methods:**

PubMed, Web of Science and Scopus databases were searched using multiple relevant keywords and screening carried out based on inclusion/exclusion criteria from March 2001 to October 2021. The random-effects model was used to conduct the meta-analysis.

**Results:**

Of the 7,377 identified articles, 56 met the inclusion criteria. Pooling all studies, the prevalence of *Clostridioides* (*Clostridium*) *difficile*, *Clostridium perfringens*, *Klebsiella oxytoca*, and *Staphylococcus aureus* as AAD-related bacteria among hospitalized patients were 19.6%, 14.9%, 27%, and 5.2%, respectively. The prevalence of all four bacteria was higher in Europe compared to other continents. The highest resistance of *C*. *difficile* was estimated to ciprofloxacin and the lowest resistances were reported to chloramphenicol, vancomycin, and metronidazole. There was no or little data on antibiotic resistance of other bacteria.

**Conclusions:**

The results of this study emphasize the need for a surveillance program, as well as timely public and hospital health measures in order to control and treat AAD infections.

## Introduction

Antibiotic-associated diarrhea (AAD) is a relatively common complication that often occurs during or after antibiotic treatment. The incidence of AAD varies by 5 to 25% depending on the type of antibiotic used [1]. It has been reported that *Clostridioides (Clostridium) difficile*, *Staphylococcus aureus*, *Clostridium perfringens*, and *Klebsiella oxytoca* as opportunistic pathogens are the predominantly bacterial agents associated with AAD [2].

*C*. *difficile* is a ubiquitous, spore-forming and gram-positive rod-shaped bacterium that produces two toxins, enterotoxin A (TcdA) and cytotoxin B (TcdB) [3, 4]. *C*. *difficile* is associated with AAD and pseudomembranous colitis (PMC) [5]. PMC is one of the most common causes of bacterial diarrhea in hospitalized patients that its incidence and mortality rate are exponentially increasing with the use of antibiotics [6]. Although this pathogen is thought to be confined to the hospitalized patients, it may be transmitted to symptomatic and asymptomatic outpatients.

Approximately 25% of AAD cases are caused by *C*. *difficile*, but it is difficult to estimate the prevalence in developing countries where knowledge, diagnostic resources and monitoring protocols are limited [7, 8]. Asymptomatic *C*. *difficile*-carriers reach 14% among hospitalized elderly patients, and 14% to 30% among antibiotic-treated individuals [9, 10]. Mortality rate associated with *C*. *difficile* antibiotic diarrhea (CDAD) are high, especially in patients above 65 years old with concomitant conditions, severe underlying disease or hypertension [11]. Other risk factors that affect the rate of mortality include the use of proton pump inhibitors, immunocompromising conditions, and prior hospitalization [7, 12, 13]. The most frequent antibiotics causing *C*. *difficile* AAD are clindamycin, fluoroquinolones, and cephalosporins, while parenteral aminoglycosides, vancomycin, and metronidazole are less frequently antibiotics involved in *C*. *difficile* infections [14].

In 1984, *Clostridium perfringens* was first reported as the cause of AAD in patients with nosocomial diarrhea. Unlike *C*. *difficile* infection, *C*. *perfringens* AAD does not result in the formation of pseudomembranes [15]. *C*. *perfringens* species are classified into seven types of A to G based on their ability to produce six major toxins [16]. It has been estimated that up to 2–15% of all AAD patients were infected with enterotoxigenic *C*. *perfringens* [17, 18]. *C*. *perfringens* enterotoxin (CPE)-positive toxinotype A (currently called *C*. *perfringens* type F strain) is considered as the most important causative agent of AAD [7, 16]. CPE is a ~35 kDa protein binds to the gut epithelial cells and, by entering the cell membrane, changes the permeability of the membrane and the loss of fluids and ions, which eventually leads to diarrhea [19, 20].

*Klebsiella oxytoca* is a gram-negative rod-shaped bacterium and an opportunistic intestinal pathogen cause of antibiotic-associated hemorrhagic colitis (AAHC). The particular form of AAHC induced by *K*. *oxytoca* performed by Koch’s postulates has received much more attention [21, 22]. This form of colitis was first described in 1978 but recently it has been shown that a cytotoxin is responsible for pathologic features of AAHC [22]. Experimentally, this cytotoxin has been shown to cause cell death of many cell lines [23]. The clinical features of AAHC differ mainly from diarrhea associated with AAD colitis. In contrast to the colitis caused by *C*. *difficile*, colitis caused by *K*. *oxytoca* is usually fragmentary and is mainly found in the right colon. The hemorrhagic diarrhea caused by *K*. *oxytoca* was mainly observed in young and outpatient individuals after short treatment with antibiotics such as amoxicillin-clavulanate, amoxicillin, penicillins and ampicillin [24]. However, *C*. *difficile*-associated diarrhea occurs mainly in elderly hospitalized patients. AAHC is characterized by sudden onset of bloody diarrhea during antibiotic treatment, usually associated with severe abdominal cramps [25, 26]. Key macroscopic feature of the AAHC is the definitive distribution of mucosal bleeding at endoscopy and mucosal examination [27]. Today, the prevalence of high-level resistance among clinical isolates of *Klebsiella* species is increasing. AAHC has also been reported after antibiotic therapy with quinolones and cephalosporins [21].

*Staphylococcus aureus* is also a less-known agent for AAD, often referred to as large-scale enterocolitis of watery diarrhea. From 1955 to 1970, *S*. *aureus* was suspected as the cause of AAD [28, 29]. Increase in the prevalence of *C*. *difficile* in recent years has led to a lack of recognition of *S*. *aureus* as a cause of nosocomial infections and AAD [30]. In contrast to food poisoning, *S*. *aureus* AAD is a gastrointestinal infection that often occurs following antibiotic-induced dysbiosis of the gut microbiota [7]. Evidence demonstrates that enterotoxin-producing strains of methicillin-resistant *S*. *aureus* may cause diarrhea associated with nosocomial antibiotics [31]. AAD-associated methicillin-resistant *S*. *aureus* strains have been reported in the blood of some patients, causing colitis to be a probable source of septicemia [32].

In early 1983, Holmberg et al. reported 18 patients with diarrhea due to multidrug-resistance *Salmonella newport* strains that were resistant to ampicillin, carbenicillin, and tetracycline. The source of infection was a hamburger eaten from an infected beef from cattle fed with subtherapeutic doses of chlortetracycline. Twelve of these patients had taken penicillin derivatives for medical complications other than diarrhea. According to reports, seems likely that these patients had an asymptomatic infection with drug-resistant *S*. *newport* before taking antibiotics for *C*. *difficile* and its toxins. Given the rarity of the disease, this is the only report in which *Salmonella* has been identified as an AAD agent [33].

In spite of the diagnosis importance of AAD-causing bacteria, it seems that less attention has been paid to these bacteria, in particular to *C*. *perfringens*, *S*. *aureus* and *K*. *oxytoca*. Also, a few number comprehensive reviews have been published in this field. Therefore, here we systematically reviewed all published articles on bacteria related to AAD in hospitalized patients from March 2001 to October 2021.

## Materials and methods

### Search strategy

Three literature databases, PubMed, Web of Science, and Scopus were used to systematically identify studies of bacterial AAD. All studies that have been published from March 2001 to October 2021 were covered. The search terms were (Antibiotic associated diarrhea OR AAD) AND (diarrhea) OR (diarrhoea) AND (Antibiotic) AND (“*Clostridioides difficile*” OR *Clostridium difficile* OR *C*. *difficile*) AND (*Clostridioides difficile* associated diarrhea OR *Clostridium difficile* associated diarrhea) AND, (“*Clostridium perfringens*” OR *C*. *perfringens*) AND (*Clostridium perfringens* antibiotic-associated diarrhea), (“*Staphylococcus aureus*” OR *S*. *aureus*) AND (*Staphylococcus aureus* antibiotic-associated diarrhea), (“*Klebsiella oxytoca*” OR *K*. *oxytoca*) AND (*Klebsiella oxytoca* antibiotic-associated diarrhea). Manual searches were performed in the reference list of retrieved articles to identify more relevant papers. Duplicates were removed using EndNote X7 (Thomson Reuters, New York, NY, USA). The PRISMA guidelines were followed to perform the study [34].

### Ethical statement

This systematic review and meta-analysis study was carried out with the Code of Ethics Committee No. IR.KUMS.REC.1398.017 adopted by Kermanshah University of Medical Sciences.

### Inclusion/Exclusion criteria and data extraction

Any cross-sectional study was included in the analysis and clinical trials, case reports, narrative and systematic reviews and meta-analysis papers were excluded. The cross sectional articles were included if they met all the following criteria: hospital related studies, frequency or prevalence of *C*. *difficile*, *C*. *perfringens*, *K*. *oxytoca*, and *S*. *aureus* among AAD, published study based on English language only, reporting laboratory-confirmed bacterial AAD. Laboratory test included: Culture, Polymerase Chain Reaction (PCR), Enzyme immunoassay (EIA), reversed passive latex agglutination test (RPLA) and enzyme-linked immunofluorescent assay (ELFA). The exclusion criteria were: 1) insufficient information on bacterial AAD, 2) articles with similar titles published in different journals. The following information were extracted from included studies: observed study, publication year, sampling year, study country, sample size, antibiotics used, history of antibiotic, prevalence /frequency of AAD, diagnostic test, and antibiotic resistance. The antibiotics that had been used in studies were applied for a random-effects model subgroup analysis. The Critical Appraisal tools of Joanna Briggs Institute (JBI) were used to perform the quality assessment (risk of bias) of each study [35].

### Bacterial prevalence in AAD patients

In each study the number of bacterial isolates has been found by culturing, toxin detection (by immunoassay or molecular methods), or both approaches. Therefore, the pooled prevalence of *C*. *difficile*, *C*. *perfringens*, *K*. *oxytoca*, and *S*. *aureus* in AAD patients was assessed based on the detection method (culturing or toxin detection) or regardless the method (total). For all meta-analyses on bacteria prevalence, the random-effects model was applied and the proportion of bacteria cases over sample size was used as effect size. In the tables and text, the proportions were multiplied by 100 to present the results by percentages.

### Limitations of study

Some limitations of this meta-analysis must be considered here. First, the distribution of studies on AAD related to bacteria is not uniform in continents and countries, and there was no published data from many countries. Second, the data concerning the age and sex of the patients as well as the exact antibiotics used or caused AAD were missing in many papers and could not be addressed or analyzed. Finally, just like many systematic review and meta-analysis papers, potential bias should be considered as a limitation.

### Statistical analysis

All analyses were performed using of version 2.2.064 of Comprehensive Meta-Analysis software. The prevalence of *C*. *difficile*, *C*. *perfringens*, *K*. *oxytoca*, and *S*. *aureus* in AAD patients, and the proportion of antibiotic resistance of each bacterium were presented with 95% confidence intervals (CIs). The random-effects model was used to conduct the meta-analysis. Based on the sampling years and the region of each study, several subgroup analyses were performed to assess the source of heterogeneity. Cochrane Q and I^2^ statistics were used to measure between studies heterogeneity. Considering the potential asymmetrical data distribution, Egger’s linear regression test was used to evaluate any publication bias. The p-value <0.05 was accepted as the statistically significance threshold.

## Results

### Study selection

**Fig 1:**
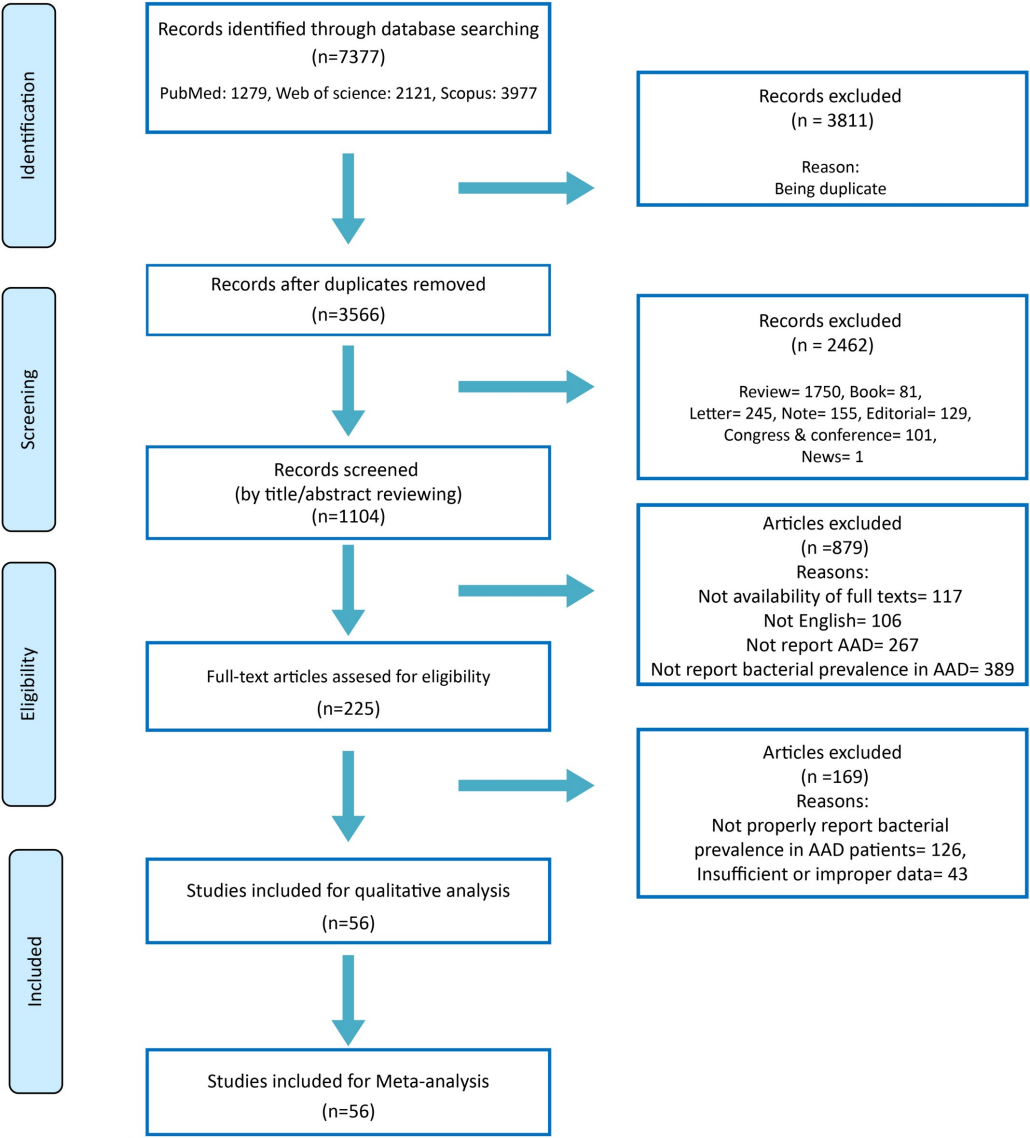
Systematic literature review flow diagram.

A total of 7,377 articles were found. After applying screening and eligibility approaches, finally, 56 full-text articles were included on basis of our criteria (Fig 1). Continental distribution of the studies was as follow: 26 studies from Asia, 15 studies from Europe, 10 studies from South America, three studies from North America and two studies from Africa. The characteristics of the final included studies are represented in Table 1.

**Table 1:** The characteristics of the studies.

Study	Published year	Sampling year	Country	Detection method	AAD No.	Isolate No. (culturing)				Isolate No. (toxin detection)				Antibiotic resistance (No.)*			Reference
						*C*. *difficile*	*C*. *perfringens*	*K*. *oxytoca*	*S*. *aureus*	*C*. *difficile*	*C*. *perfringens*	*K*. *oxytoca*	*S*. *aureus*	*C*. *difficile*	*K*. *oxytoca*	*S*. *aureus*	
**Abrahao et al.**	2001	1998	Germany	ELISA, Culture	156					10	10						[40]
**Ackermann et al.**	2005	2002–2003	Germany	EIA, Culture, PCR	89	39	5	25		26				FUS(0), LZD(1), MTZ(0), TET(10), VAN(0)		OXA(0)	[37]
**Alikhani et al.**	2016	2011–2013	Iran	Culture, PCR	331				57				40		AMK(1), AMP(32), AMX(34), ETP(1), IPM(3), MEM(4), TIC(22)		[41]
**Alinejad et al.**	2015	2013–2014	Iran	EIA	37					8							[42]
**Al-Tawfiq et al.**	2010	2007–2008	Saudi Arabia	ELISA	913					42							[43]
**Asha et al.-1**	2002		UK	ELISA, Culture, PCR	200		74			32	16						[44]
**Asha et al.-2**	2006	2001–2002	UK	ELISA, Culture, PCR	735			10		591	155	8				MET(10)	[17]
**Azimirad et al.**	2019	2011–2017	Iran	ELISA, Culture, PCR	303		68										[38]
**Balassiano et al.**	2010	2006–2009	Brazil	ELISA, Culture, PCR	218					43							[45]
**Bishara et al.**	2008	1999–2000	Israel	EIA	217					52							[46]
**Cancado et al.**	2018	2011–2015	Brazil	EIA, Culture, PCR	154	44				34							[47]
**Chaudhry et al.**	2008	2001–2005	India	ELISA, Culture, PCR	524		1			37				CHL(0), CLI(0), ERY(0), MTZ(0), PEN(0), TET(10), VAN(0)			[48]
**Dai et al.**	2020	2014–2016	China	ELFA, Culture, PCR	122	55				38				CLI(34), ERY(48), LVX(8), MTZ(0), RIF(7), TGC(0), VAN(0)			[49]
**Djebbar et al.**	2018	2013–2015	Algeria	Culture, PCR	159	11				7				AMK(0), CIP(11), CLI(2), ERY(2), MTZ(0), MXF(0), VAN(0)			[50]
**Elseviers et al.**	2015		Belgium	Culture	71	4											[51]
**Ergen et al.**	2009	2004–2005	Turkey	EIA, Culture, PCR	44	19								CIP(19), CLI(0), ERY(0), MTZ(0), MXF(0), TET(0), VAN(0)			[52]
**Farshad et al.**	2013	2012	Iran	EIA, Culture	122	9											[53]
**Ferreira et al.**	2003	2000–2001	Brazil	Culture, PCR	18	5											[54]
**Haran et al.-1**	2014	2012–2013	USA	EIA	45					2							[55]
**Haran et al.-2**	2016	2013	USA	ELISA	275					52							[56]
**Hassan et al.**	2012	2008	Malaysia	EIA	105					24							[57]
**Heimesaat et al.**	2005	2003	Germany	ELISA, Culture, PCR	693	83	147			79	1						[58]
**Hogenauer et al.**	2006	2001–2004	Austria	Culture	6				5								[21]
**Ingle et al.**	2013	2009–2010	India	ELFA	150					12							[59]
**Kim et al.**	2017		Korea	EIA, PCR	135					26	14						[60]
**Kumar et al.**	2014		India	EIA, Culture	273	3				9							[61]
**Lee et al.**	2012	2009–2010	Taiwan	EIA, Culture	80					8							[62]
**Legaria et al.**	2003	2000–2001	Argentina	EIA, Culture	87	32											[63]
**Li et al.**	2016	2008–2010	China	ELFA, Culture	470					93							[64]
**Lv et al.**	2014	2008–2010	China	ELFA	130					45							[65]
**Maestri et al.**	2020	2017–2019	Brazil	EIA, PCR	351					62							[66]
**Mane et al.-1**	2021	2017–2019	India	Culture, ELISA	222	20				70							[67]
**Mane et al.-2**	2020		India	PCR	222					18							[68]
**Martirosian et al.**	2005	2001–2002	Poland	ELISA, Culture, PCR	56	18				12				CLI(4), ERY(4)			[69]
**Mirzaei et al.**	2018		Iran	Culture, PCR	100	8				2							[70]
**Naaber et al.**	2011		Norway	Culture, PCR	74	42				59							[71]
**Naqvi et al.**	2012	2002–2009	Pakistan	Culture	473	191											[72]
**Pinto et al.**	2003		Brazil	Culture	210	14				16							[73]
**Pituch et al.**	2007	2004–2005	Poland	EIA, Culture, PCR	52					39	21						[74]
**Plaza-Garrido et al.**	2016	2011–2012	Chile	Culture, PCR	392	81											[75]
**Rodriguez-Varon et al.**	2017	2014–2015	Colombia	PCR	43					6				VAN(0)			[76]
**Sachu et al.**	2018	2014–2017	India	ELFA	660					64							[77]
**Sadeghifard et al.**	2010	2002–2006	Iran	Culture	942	57								CFP(10), CHL(0), CIP(37), CLI(49), CRO(0), CST(57), FEP(11), GEN(57), KAN(57), MTZ(5), TET(17), VAN(0)			[78]
**Secco et al.**	2014	2009–2010	Brazil	ELISA, Culture, PCR	74	3				3				CIP(4), LVX(4), MTZ(0), MXF(1), VAN(0)			[79]
**Shaheen et al.**	2007		Egypt	EIA, Culture, PCR	150					36	18						[80]
**Song et al.**	2008	2005	Korea	ELISA, Culture	38	4		1		5							[81]
**Spadao et al.**	2014	2007–2011	Brazil	Culture	64					9							[82]
**Vaishnavi et al.**	2005	2000–2002	India	ELISA, RPLA, Culture	239	47	23										[83]
**Wistrom et al.**	2001	1995–1996	Sweden	ELISA	83					46							[84]
**Wong et al.**	2017	2014–2015	UK	EIA	32					2							[85]
**Yilmaz et al.**	2012	2006	Turkey	ELISA, Culture	21					6			11				[86]
**Zarandi et al.**	2017	2014–2015	Iran	ELISA, Culture, PCR	233	49				25							[87]
**Zhao et al.**	2020	2011–2014	China	PCR	197					84							[88]
**Zhou et al.**	2014	2012–2013	China	EIA, Culture, PCR	206	63											[89]
**Zollner-Schwetz et al.**	2008	2006–2008	Austria	Culture	107				4								[90]
**Zumbado-Salas et al.**	2008		Costa Rica	EIA, Culture, PCR	104	35				31							[91]

**Abbreviations**: AAD: antibiotic-associated diarrhea, AMK: Amikacin, AMP: Ampicillin, AMX: Amoxicillin, CFP: Cefoperazone, CHL: Chloramphenicol, CIP: Ciprofloxacin, CLI: Clindamycin, CRO: Ceftriaxone, CST: Colistin, ERY: Erythromycin, ETP: Ertapenem, FEP: Cefepime, FUS: Fusidic acid, GEN: Gentamicin, IPM: Imipenem, KAN: Kanamycin, LZD: Linezolid, LVX: Levofloxacin, MEM: Meropenem, MET: Methicillin, MTZ: Metronidazole, MXF: Moxifloxacin, OXA: Oxacillin, PEN: Penicillin, RIF: Rifampin, TET: Tetracycline, TIC: Ticarcillin, TGC: Tigecycline, VAN: Vancomycin, EIA: enzyme immunoassay, ELISA: enzyme-linked immunosorbent assay, ELFA: enzyme-linked immunofluorescent assay, PCR: polymerase chain reaction, RPLA: reversed passive latex agglutination test

*: No study reported antibiotic resistance of *C*. *perfringens*

**Note**: If a study had tested previously confirmed isolates by another method, only previously confirmed isolates (that were contributed to the the prevalence in whole AAD populations) were reported here.

### Bacterial prevalence in AAD patients

#### *C*. *difficile* prevalence in AAD patients

**Fig 2:**
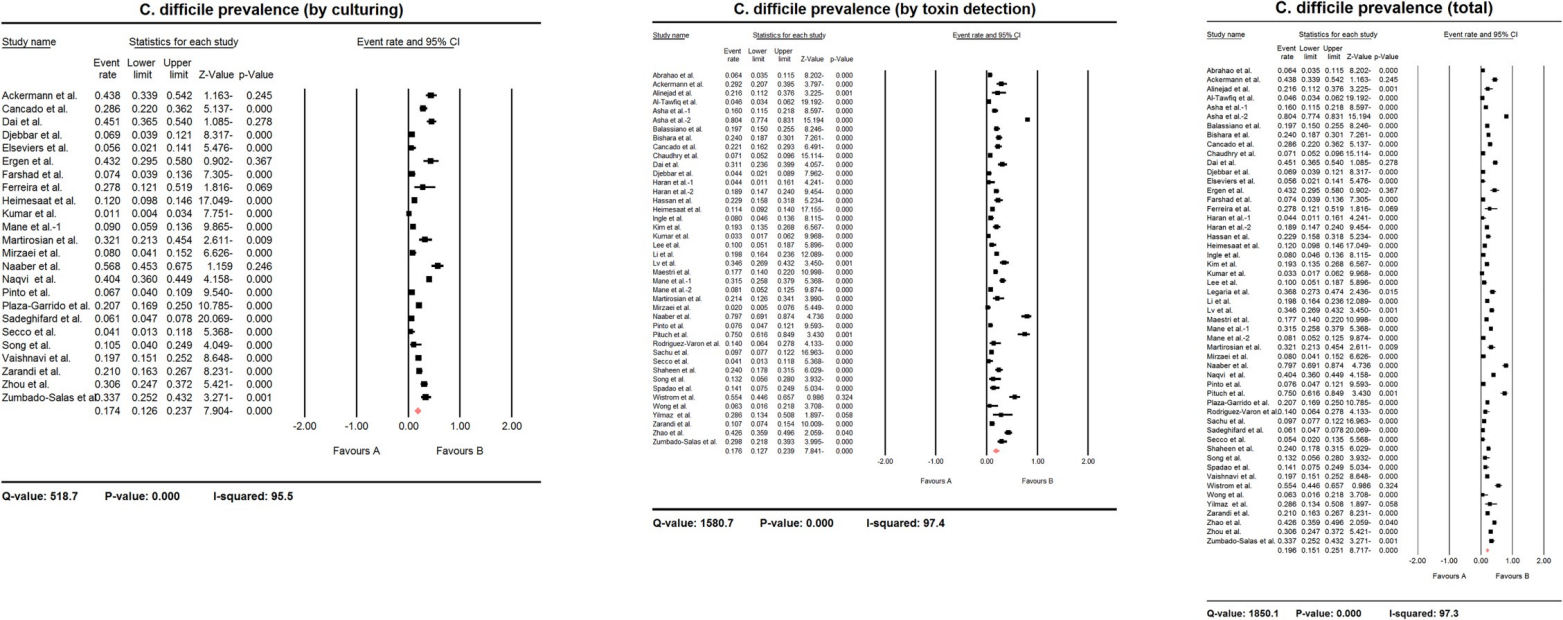
The forest plots of prevalence of *C*. *difficile* in AAD patients. The plots indicate the estimated pooled prevalence of *C*. *difficile* in AAD patient based on culturing, toxin detection or regardless the detection method (total). The heterogeneity test results are shown below each plot.

Regardless the detection method, 52 studies were applied for meta-analysis, in which the pooled prevalence of *C*. *difficile* in AAD patients was 19.6% (CI 95%: 15.1–25.1). Considering culturing method, 24 studies were applied for meta-analysis, in which the pooled prevalence of *C*. *difficile* in AAD patients was 17.4% (CI 95%: 12.6–23.7). Based on toxin detection methods, 42 studies were applied for meta-analysis, in which the pooled prevalence of *C*. *difficile* in AAD patients was 17.6% (CI 95%: 12.7–23.9) (Fig 2).

In all three meta-analyses on *C*. *difficile* prevalence in AAD patients the confidence intervals of summary effect did not include zero and the null hypothesis was rejected, meaning that there was a positive prevalence of *C*. *difficile* in AAD patients. Also, in all analyses, the Q-values were much more than the number of studies minus 1 (degrees of freedom) indicating a significant heterogeneity between studies. The I^2^ statistics showed that a range of 95.5 to 97.4% of the variances in the observed effects is because of variances in the true effects (Fig 2).

#### *C*. *perfringens* prevalence in AAD patients

**Fig 3:**
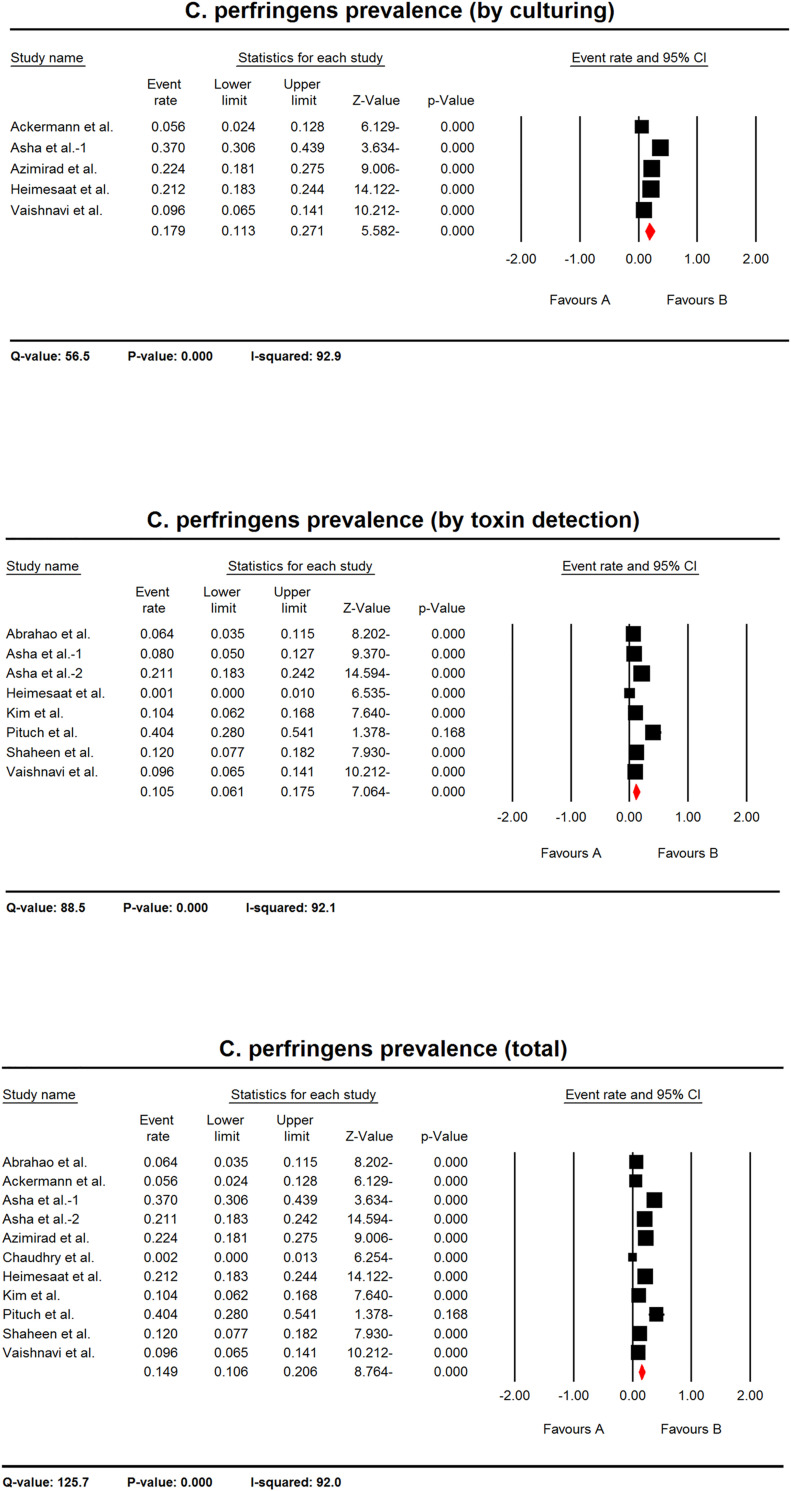
The forest plots of prevalence of *C*. *perfringens* in AAD patients. The plots show the estimated pooled prevalence of *C*. *perfringens* in AAD patient based on culturing, toxin detection or regardless the detection method (total). The heterogeneity test results are shown below each plot.

Eleven studies were used for meta-analysis regardless the detection method, in which the pooled prevalence of *C*. *perfringens* in AAD patients was 14.9% (CI 95%: 10.6–20.6). Five studies were applied for meta-analysis based on culturing method in which the pooled prevalence of *C*. *perfringens* in AAD patients was 17.9% (CI 95%: 11.3–27.1). Considering toxin detection methods, eight studies were applied for meta-analysis, in which the pooled prevalence of *C*. *perfringens* in AAD patients was 10.5% (CI 95%: 6.1–17.5) (Fig 3).

The confidence intervals of summary effect in all analyses did not include zero, hence rejecting the null hypothesis and showed that there was a positive prevalence of *C*. *perfringens* in AAD patients. The Q-values in all analyses were much more than degrees of freedom showing a significant heterogeneity between studies. The I^2^ statistics showed that a range of 92.0 to 92.9% of the variances in the observed effects is because of variances in the true effects (Fig 3).

#### *K*. *oxytoca* prevalence in AAD patients

**Fig 4:**
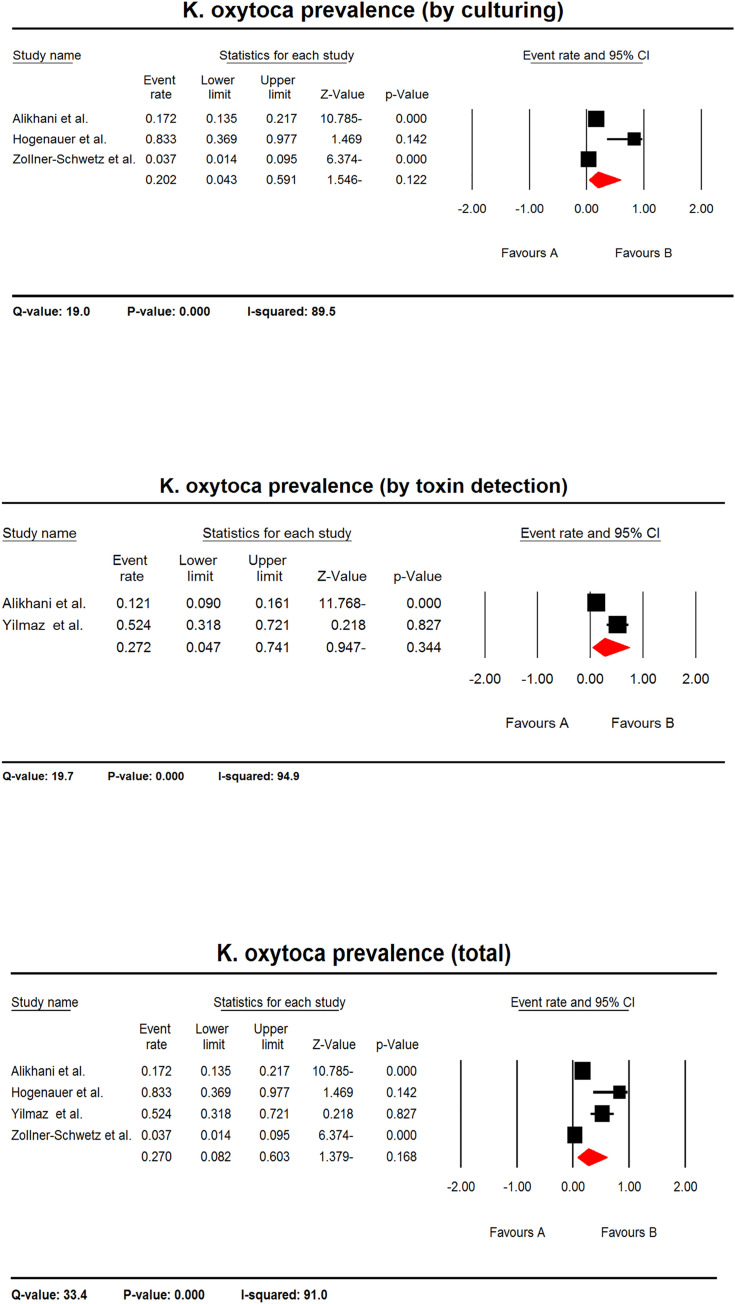
The forest plots of prevalence of *K*. *oxytoca* in AAD patients. The plots represent the estimated pooled prevalence of *K*. *oxytoca* in AAD patient based on culturing, toxin detection or regardless the detection method (total). The heterogeneity test results are shown below each plot.

The pooled prevalence of *K*. *oxytoca* in AAD patients was 27.0% (CI 95%: 8.2–60.3) in four studies included, regardless the detection method. Based on culturing method, three studies were applied for meta-analysis, in which the pooled prevalence of *K*. *oxytoca* in AAD patients was 20.2% (CI 95%: 4.3–59.1). Regarding toxin detection methods, two studies were used for meta-analysis, in which the pooled prevalence of *K*. *oxytoca* in AAD patients was 27.2% (CI 95%: 4.7–74.1) (Fig 4).

Similar to other bacteria, there was a positive prevalence of *K*. *oxytoca* in AAD patients since the confidence intervals of summary effect did not include zero in all three meta-analyses. Also, the Q-values indicated a significant heterogeneity between studies. The I^2^ statistics showed that a range of 89.5 to 94.9% of the variances in the observed effects is because of variances in the true effects (Fig 4).

#### *S*. *aureus* prevalence in AAD patients

**Fig 5:**
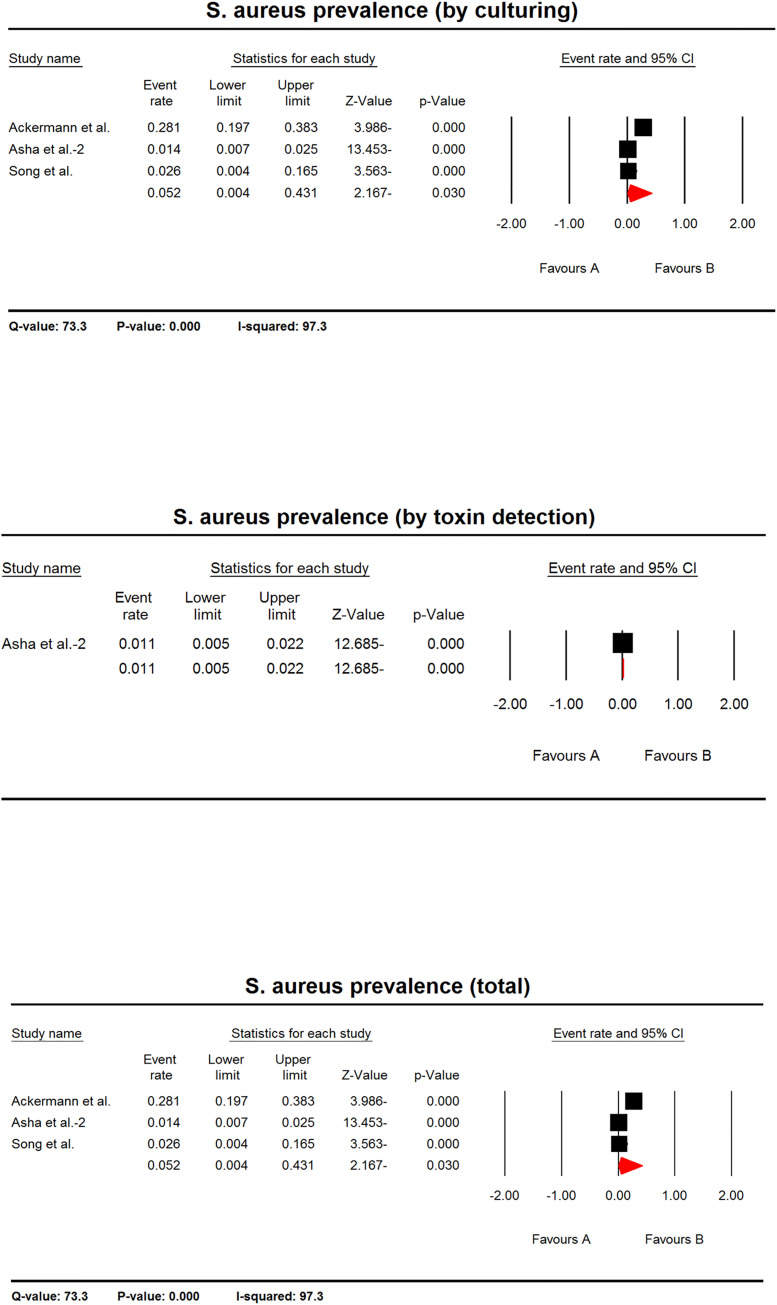
The forest plots of prevalence of *S*. *aureus* in AAD patients. The plots indicate the estimated pooled prevalence of *S*. *aureus* in AAD patient based on culturing, toxin detection or regardless the detection method (total). The heterogeneity test results are shown below each plot. Only one study included in analysis based on toxin detection, thus the heterogeneity test was not applicable for it.

In another meta-analysis using three studies and regardless the detection method, the pooled prevalence of *S*. *aureus* in AAD patients was 5.2% (CI 95%: 0.4–43.1). Considering culturing method, three studies were applied for meta-analysis, in which the pooled prevalence of *S*. *aureus* in AAD patients was 5.2% (CI 95%: 0.4–43.1). Regarding toxin detection methods, one study was applied for meta-analysis, in which the pooled prevalence of *S*. *aureus* in AAD patients was 1.1% (CI 95%: 0.5–2.2) (Fig 5).

The null hypothesis was rejected in all meta-analyses on *S*. *aureus* prevalence in AAD patients meaning that there was a positive prevalence of *S*. *aureus* in AAD patients. Also, in analyses regardless methods and that based on culturing, the Q-values were more than the degrees of freedom indicating a significant heterogeneity between studies. Only one study included in analysis based on toxin detection, thus the heterogeneity test was not applicable for it. Based on I2 statistics, 97.3% of the variances in the observed effects based on culturing and regardless the detection method is because of variances in the true effects (Fig 5).

### Subgroup analysis of bacterial prevalence in AAD patients based on the sampling year

To subgroup analysis of the bacterial prevalence in AAD patients based on the sampling year, the studies were divided into three groups as follow: D1 (≤2000), D2 (2001–2005), D3 (2006–2010), D4 (2011–2015), and D5 (2016≤). Based on these times, 38, 7, 4, and 3 studies were used for subgroup analysis on the prevalence of *C*. *difficile*, *C*. *perfringens*, *K*. *oxytoca*, and *S*. *aureus* in AAD patients, respectively. Subgroup analyses were done through the random-effects model on the total prevalence of bacteria (regardless the detection methods). For *C*. *perfringens*, *K*. *oxytoca*, *and S*. *aureus* the studies were divided into only one group and other groups included no or only one study. Therefore the statistical comparison of the bacterial prevalence between time subgroups was not accurately applicable for these three bacteria. The prevalence of *C*. *difficile* was decreased after 2006 onward, although there was not significant heterogeneity between subgroups (Q-value: 4.808, p-value: 0.308) (Table 2).

**Table 2:** Subgroup analysis of bacterial prevalence in AAD patients based on the sampling year.

Group name	Sampling year	Number of studies	Prevalence (%)	Lower limit	Upper limit	Z-value	*p*-value
***C*. *difficile***							
**D1**	≤2000	3	23.4	7.2	53.8	-1.70	0.082
**D2**	2001–2005	11	32.5	19.2	49.3	-2.04	0.042
**D3**	2006–2010	9	14.4	7.2	26.9	-4.47	0.000
**D4**	2011–2015	12	16.6	9.2	28.3	-4.62	0.000
**D5**	2016≤	3	23.9	5.9	61.2	-1.41	0.160
**Overall**	-	38	20.9	15.3	28	-6.76	0.000
Test of heterogeneity between subgroups: Q-value: 4.808, p-value: 0.308							
***C*. *perfringens***							
**D1**	≤2000	1	6.4	1.8	20.0	-4.05	0.000
**D2**	2001–2005	6	14.1	8.8	21.8	-6.65	0.000
**Overall**	-	7	12.6	8.1	19.1	-7.69	0.000
Test of heterogeneity between subgroups: Q-value: 1.489, p-value: 0.222							
***K*. *oxytoca***							
**D2**	2001–2005	1	83.3	3.2	99.9	0.63	0.530
**D3**	2006–2010	2	17.3	0.8	84.7	-0.94	0.349
**D4**	2011–2015	1	17.2	0.2	95.2	-0.68	0.499
**Overall**	-	4	29.5	3.8	81.4	-0.73	0.467
Test of heterogeneity between subgroups: Q-value: 1.201, p-value: 0.549							
***S*. *aureus***							
**D2**	2001–2005	3	0.05	0.00	0.43	-2.17	0.030
**Overall**	-	3	0.05	0.00	0.43	-2.17	0.030

Test of heterogeneity between subgroups: Q-value: 0.000, p-value: 1.000

#### Subgroup analysis of bacterial prevalence in AAD patients based on the region

To subgroup analysis of the bacterial prevalence in AAD patients based on the region, the studies were divided into five groups as follow: Africa, Asia, Europe, North America, and South America. Based on these regions, 52, 11, 4, and 3 studies were used for subgroup analysis on the prevalence of *C*. *difficile*, *C*. *perfringens*, *K*. *oxytoca*, and *S*. *aureus* in AAD patients, respectively. The prevalence of all four bacteria was higher in Europe compared to other continents, although there was not significant heterogeneity between subgroups (Table 3).

**Table 3:** Subgroup analysis of bacterial prevalence in AAD patients based on the continents.

Group name	Number of studies	Prevalence (%)	Lower limit	Upper limit	Z-value	*p*-value
***C*. *difficile***						
**Africa**	2	13.5	3.2	42.1	-2.36	0.018
**Asia**	23	15.7	10.5	22.6	-7.28	0.000
**Europe**	14	32.5	21.0	46.5	-2.42	0.015
**North America**	3	16.9	5.2	42.8	-2.39	0.017
**South America**	10	17.5	9.5	29.9	-4.35	0.000
**Overall**	52	19.6	15.3	24.9	-9.06	0.000
Test of heterogeneity between subgroups: Q-value: 6.999, p-value: 0.136						
***C*. *perfringens***						
**Africa**	1	12.0	3.6	33.2	-3.01	0.003
**Asia**	4	9.4	4.9	17.3	-6.34	0.000
**Europe**	6	19.5	12.5	29.1	-5.25	0.000
**Overall**	11	14.8	10.4	20.6	-8.56	0.000
Test of heterogeneity between subgroups: Q-value: 3.740, p-value: 0.154						
***K*. *oxytoca***						
**Asia**	1	17.2	0.2	94.9	-0.68	0.494
**Europe**	3	35.0	3.4	89.1	-0.45	0.655
**Overall**	4	29.4	3.9	81.0	-0.74	0.462
Test of heterogeneity between subgroups: Q-value: 0.125, p-value: 0.723						
***S*. *aureus***						
**Asia**	1	2.6	0.0	80.2	-1.41	0.158
**Europe**	2	6.9	0.3	66.2	-1.56	0.119
**Overall**	3	5.2	0.4	45.9	-2.08	0.038

Test of heterogeneity between subgroups: Q-value: 0.108, p-value: 0.742

### The bacteria antibiotics susceptibility in AAD patients

A few number of studies were on *C*. *perfringens*, *K*. *oxytoca*, and *S*. *aureus* antibiotics susceptibility in AAD patients (Table 1). Therefore, the meta-analysis on antibiotics susceptibility was only done for *C*. *difficile*. The antibiotic susceptibility have been reported in at least two articles. These antibiotics included chloramphenicol (CHL), ciprofloxacin (CIP), clindamycin (CLI), erythromycin (ERY), levofloxacin (LVX), metronidazole (MTZ), moxifloxacin (MXF), tetracycline (TET), and vancomycin (VAN) (Table 4).

**Table 4:** The percentages of antibiotics resistance of *C*. *difficile* AAD.

Antibiotic	Number of studies	Resistance cases (%)	Lower limit (%)	Upper limit (%)	Z-value	*p*-value
**CHL**	2	1.6	0.1	20.2	-2.95	0.003
**CIP**	4	88.4	57.6	97.7	2.31	0.021
**CLI**	6	31.8	11.8	62.0	-1.19	0.233
**ERY**	5	25.3	7.6	58.3	-1.50	0.134
**LVX**	2	38.3	6.2	85.4	-0.42	0.678
**MTZ**	7	3.6	0.9	12.8	-4.71	0.000
**MXF**	3	7.4	0.9	40.7	-2.31	0.021
**TET**	4	14.0	3.3	43.8	-2.27	0.023
**VAN**	8	2.6	0.7	9.5	-5.19	0.000
**Overall**	41	15.4	9.6	23.7	-6.25	0.000

Test of heterogeneity between subgroups: Q-value: 38.37, p-value: 0.000

The highest resistance of *C*. *difficile* were estimated to CIP (88.4%, CI 95%: 57.6–97.7) and the lowest resistances were reported to CHL (1.6%, CI 95%: 0.1–2.0), VAN (2.6%, CI 95%: 0.7–9.5), and MTZ (3.6%, CI 95%: 0.9–12.8) (Table 4). There was a significant heterogeneity between subgroups (Q-value: 38.37, p-value: 0.000) (Table 4).

### Publication bias

To assess the publication bias, the prevalence of *C*. *difficile* in AAD patients regardless the detection methods was applied. The Egger’s test showed a significant publication bias in the reports of *C*. *difficile* prevalence in AAD patients (p-value = 0.03).

## Discussion

The studies on bacteria associated with AAD are limited. Therefore, here we collected all published data on bacteria associated with AAD including *C*. *difficile*, *C*. *perfringens*, *K*.*oxytoca*, and *S*. *aureus*.

*C*. *difficile* is known to be the most important cause of AAD in the world. Using various databases, we found 52 articles about the *C*. *difficile* AAD data. The pooled prevalence of *C*. *difficile* among hospitalized patients with AAD was 19.6%. These prevalence is similar to that was previously published in a systematic review and meta-analysis by Nasiri et al. (20%) [36], but it was a little different from study by Curcio et al. about *C*. *difficile* AAD in developing countries (15%) [8]. The prevalence of *C*. *difficile* AAD varies in different continents so that the highest prevalence of *C*. *difficile* AAD was in Europe (32.5%) and the lowest frequency was in Africa (13.5%).This difference can be attributed to various factors including large population migrations, and appropriate program monitoring about *C*. *difficile* AAD in Europe than other countries.

The prevalence of *C*. *difficile* has decreased after 2006 onward, which could be due to increasing in world health state, more proper prescription, and increasing in general awareness of the adverse effects of antibiotic overuse. However, inappropriate use of antibiotics is constantly continued nowadays, although the emergence of drug-resistant bacteria cause real concerns in the world. In order to prevent the spread of resistant isolates and bacterial infections, continuous monitoring of how the antibiotic resistance of bacteria appears is essential.

Based on the meta-analysis, the percentages of antibiotics resistance of *C*. *difficile* AAD was high for CIP and low for CHL, VAN and MTZ that were in concordant with Nasiri [36] and Ackermann [37] studies. Although, the frequency of resistance to first line antibiotics to treat AAD (MTZ and VAN) is still low, there is a concern to increase this rate in the future due to overuse and inappropriate use of antibiotics.

Accurate and on-time diagnosis of AAD-related bacteria assists in controlling of *C*. *difficile* transmission in communities and medical centers, as well as reducing the prevalence of AAD. There are several methods for identifying *C*. *difficile* AAD or their toxins, among them culture and ELISA are used mostly. These techniques were also the most frequent methods used in our included studies. More studies are needed to compare different techniques of detection of AAD-causing bacteria.

Due to the limited or scattered information obtained from the included studies, variables such as age, sex, and the exact prevalence of antibiotics used before AAD incidence could not be analyzed in our systematic review.

*C*. *perfringens* is another bacterium that can cause disease through hospital transmission. This bacterium is a part of the gut flora in healthy humans, however its colonizing and overgrown can cause severe AAD if allowed a longer period of growth [38]. Our study seems to be the first systematic review related to *C*. *perfringens* AAD, in which the mean frequency of *C*. *perfringens* among AAD hospitalized patients was 14.9%.

Distribution of the studies in patients with *C*. *perfringens* AAD according to continent was as follows: 9.4% in Asia, 19.5% in Europe, and 12% in Africa. No study of *C*. *perfringens* AAD was found in other parts of the world, which may be due to the limited number of published articles or the lack of its correct laboratory diagnostic methods.

Like *C*. *difficile*, culture and ELISA were the most diagnostic methods used to detect *C*. *perfringens* AAD or their toxins. These techniques are able to detect *C*. *perfringens* correctly if used appropriately by experienced technicians, although other methods may also be useful in detecting of this bacterium.

Only three studies have been reported the prevalence of *S*. *aureus* in AAD patients, in them the pooled prevalence of *S*. *aureus* was low (5.2%). This prevalence is not very reliable due to the low number of published studies in this field. Likewise, routine diagnosis of *S*. *aureus* in AAD cases does not seem to be justified. Therefore, more studies are needed to find the true prevalence of *S*. *aureus* in AAD patients.

*K*. *oxytoca*, known to cause AAHC, is a distinct form of AAD. This pathogen acts as a pathobion in the human intestinal microbiota of dysbiotic and causes AAHC [39]. Our study was the first systematic review in *K*. *oxytoca* AAD. Using different databases, only four articles were finally included. The pooled prevalence was 27% and Europe (35%) and Asia (14%) were the highest and the lowest prevalence continents, respectively. Due to the limited information presented in these articles, we could not provide accurate information on *K*. *oxytoca* frequency in AAD patients.

We detected a significant heterogeneity between studies, showing that the bacterial prevalence in AAD patients is significantly different in various countries. This difference could be attributed to the quality of the studies, the sample sizes, the efficiency of diagnosis methods, or the true different distribution of bacterial causing AAD in different parts of the world.

## Conclusion

Limited studies have been reported on the most important bacteria related to AAD in different countries of the world, which may be due to lack of proper laboratory diagnostic tests. The analysis of the studies indicated that *K*. *oxytoca*, *C*. *difficile* and *C*. *perfringens* are the most prevalent among hospitalized patients with AAD in the world. The prevalence of all four bacteria was higher in Europe compared to other continents. The highest resistance of *C*. *difficile* was estimated to ciprofloxacin and the lowest resistances were reported to chloramphenicol, vancomycin, and metronidazole. There was a little data on antibiotic resistance of other bacteria. Therefore, the results of this study emphasize the need for a surveillance program, as well as timely public and hospital health measures in order to control and treat AAD infections.

## Supporting information

S1 File (XLSX)
